# Is cardiopulmonary bypass standby still required for laser lead extractions?

**DOI:** 10.1186/s13019-022-01987-4

**Published:** 2022-09-15

**Authors:** Lindsay Volk, Nina Verghis, Hirohisa Ikegami, Manabu Takebe, Mark J. Russo, Leonard Y. Lee, Anthony Lemaire

**Affiliations:** Department of Cardiothoracic Surgery, Rutgers Robert Wood Johnson Medical School, 125 Paterson Street, New Brunswick, NJ 08903 USA

**Keywords:** Laser, Lead extraction, Cardiopulmonary bypass, And cardiac surgeon

## Abstract

**Objective:**

Over the last two decades there has been an increase in the number of cardiac implantable electronic devices and consequently, there has also been an increased need for lead extractions. Fibrotic attachments develop between the lead and the venous and cardiac structures that may require the use of a laser to mobilize the lead. Cardiothoracic surgeons (CTS) have traditionally provided backup for surgical emergencies for these extractions. This study evaluates the surgical outcomes of patients undergoing transvenous laser lead extractions (TLE) and determines if CTS are still needed for backup.

**Methods:**

A retrospective review of consecutive patients undergoing laser lead extractions at a single academic center. Lead extractions using only laser sheaths were analyzed. The clinical characteristics, complications, and mortality of the patients were evaluated.

**Results:**

One hundred and twenty-one patients underwent TLEs from January 1st, 2014 to December 31st, 2018. The majority were male (N = 80, 66.1%), and the average age was 66.48 ± 14 years. The indication for removal was either laser lead malfunction or infection. A total of 30 patients (24.8%) had complications postoperatively including wound hematomas, superficial infections, and arrhythmias. The average length of stay was 9 ± 12 for all the patients in the study. 2 patients (1.6%) had injuries that required emergency surgical repair with injuries to the posterior superior vena cava and right ventricle. Both patients survived the initial injury with one patient was discharged home on day 4 and the other succumbing to his injuries on postoperative day 20.

**Conclusion:**

Although the incidence of surgical emergencies is rare the morbidity and mortality for TLE require that surgical backup be available.

## Introduction

The number of cardiovascular implantable electronic devices has increased with estimates of 1.2 to 1.4 million implanted worldwide each year [1]. As a result, of the greater number of devices, more of them will require removal over time. The most common indications for lead removal are infection, lead-lead interactions, and manufacturer recalls [2]. These extractions are not without difficulty as fibrotic attachments can develop between the lead and patient’s tissues, including the innominate vein, superior vena cava, right atrium, and ventricle. The extraction of laser leads was traditionally a very dangerous and difficult procedure that often required open surgical removal. This paradigm changed in 1999 with the publication of the PLEXES trial, which demonstrated significant clinical advantages of the use of laser tools for removal of these fibrotic leads, but also demonstrated that this method is associated with significant, even life-threatening risks [3].

Currently, removal of pacemaker leads are primarily performed by interventional cardiologists with cardiothoracic surgeons providing backup for surgical emergencies. There is a small percentage of patients who have the leads removed by cardiothoracic surgeons. One of the most recent studies showed that extractions were performed by cardiac surgeons at 20% of medical centers. Furthermore, a quarter of the operations are done without a cardiothoracic surgeon present [4]. Serious complications for laser assisted lead extraction have been well described and range from 1.9 to 3.4% in other series [5–7]. The purpose of this study was to review a contemporary cohort at a large academic medical center and determine if there is a continued need for cardiothoracic surgeon presence for these procedures.

### Methods

We present a retrospective review of consecutive patients undergoing laser lead extractions at a single academic center from January 1st, 2014 to December 31st, 2018. Cardiothoracic surgeons were available for each case. All laser lead extractions took place in the operating room under full general anesthesia and respiratory support. The anesthesiologist then prepare the patient as though they could require open-heart surgery. The patients had central venous catheters placed as well as arterial lines placed for continous blood pressure monitoring. The institutional protocol is that all patients are typed and screened prior to the procedure with blood available for immediate transfusion. A cardiopulmonary bypass circuit and perfusion team are available and on standby. A transesophageal echocardiography probe is in the room and used to evaluate the patient before the procedure begins to assess the patient’s heart function as well as to determine if a pericardial effusion is present. All procedures are performed using fluoroscopic guidance.

The clinical characteristics, complications, and mortality of the patients were evaluated. Demographic data included age, gender, and race. Relevant past medical history, preoperative and postoperative diagnosis, and the American Society of Anesthesiologists (ASA) Physical Status Classification System designation for each patient was collected. The reason for the extraction, hospital length of stay, intraoperative and postoperative complications, and mortality were included. The type of complication was described, and the number of cases that required open-heart surgery was tabulated. The results are presented as frequencies and percentages. Where applicable, continuous data is presented as mean and standard deviation.

## Results

One hundred and twenty-one patients underwent laser lead extractions from January 1st, 2014 to December 31st, 2018. Most of the patients were male (N = 80, 66.1%), with an average age of 66.48 ± 14 years. The majority of the patients underwent elective removal (n = 108, 89.3%), while 13 patients (10.7%) required urgent surgery. The most common indications for extraction were infection including lead infection, bacteremia, and valve endocarditis (52%) and lead fracture (17%). The majority of our patients were white (62%). The most common comorbidities included hypertension (65%), cardiomyopathy (61%), and coronary artery disease (41%). Demographic and preoperative factors are detailed in Table 1.

**Table 1:** Baseline characteristics

n = 121		
Demographics	Age at time of procedure (year)	66 ± 14
	Male gender, n (%)	80 (66)
	White	74 (62)
	Black	19 (16)
	Asian	7 (5.8)
	Other	21 (17)
Medical history, n (%)	Diabetes	43 (36)
	Coronary artery disease	50 (41)
	Hypertension	79 (65)
	Congestive heart failure	44 (36)
	Hyperlipidemia	35 (29)
	Atrial fibrillation	40 (33)
	CABG	22 (18)
	Cardiomyopathy (nonischemic and ischemic)	74 (61)
	Myocardial infarction	17 (14)
Primary indication for laser lead extraction	Infection (lead, bacteremia, endocarditis)	63 (52)
	Fracture	20 (17)
	Vegetation	1 (0.8)
	Dysrhythmia	15 (12)
	Malfunction (lead, device)	13 (11)
	Mechanical (recall, end of pacemaker life, upgrade)	10 (8.3)

A total of 30 patients (24.8%) had complications postoperatively including wound hematomas, superficial infections, and arrhythmias. Those with repeated incidence are detailed in Table 2. Some of the other more serious complications, each with only one incidence, included a cerebrovascular accident, a patient requiring a transfusion, and an intraoperative mortality. The average length of stay was 9 ± 12 for all the patients in the study. There were 8 mortalities (6.6%). Two patients (1.6%) had injuries that required emergency surgical repair. The posterior superior vena cava was injured in one case and right ventricle in the other. Both patients survived the initial injury and subsequent surgery, with one patient being discharged home on day 4 and one patient succumbing to his injuries on postoperative day 20. The indication for removal was laser lead malfunction in the first case and infection in the latter.

**Table 2:** Complications

Complications, n = 121	
Complication	N (%)
Acute kidney injury	3 (2.5)
Hematoma	6 (5.0)
Bradycardia	2 (1.7)
Tachycardia	2 (1.7)
Lead issue (retained remnant, take back for retracted atrial lead)	2 (1.7)

## Discussion

The data from the study shows that the incidence of complications associated with laser lead extractions were low but the morbidity and mortality is high. We found a relatively low incidence, 2.4%, of complications that required surgical intervention. The findings are consistent with the limited data in the literature [5–7]. Notably, some studies have found complication rates of less than 2% [2, 7] and it is likely that the slightly higher rate seen in this study is due to case mix given that our institution is a large academic medical center with referrals for more complicated patients. Additionally, the rates of lead extraction complications increase with patient age and comorbidities [8, 9]. Given the significant rates of comorbidities in our patient population this slightly higher complication rate can be expected. The two injuries that required surgical repair in our series—perforations to the superior vena cava and right ventricle are serious and life threatening. Both of these injuries have been reported previously and are known possible complications of laser lead extraction [10, 11]. These injuries would likely have been fatal if not for the intervention of an immediately available cardiothoracic surgeon. This highlights that even though the indications for surgical intervention are varied and rare, they are uniformly serious and life-threatening. Most complications can be repaired rapidly enough to be lifesaving, but this can only be done if personnel and equipment is immediately available. The findings of our study support the continued involvement of a cardiothoracic surgeons in laser lead extractions in order to maximize patient safety and limit mortality.

The severity of the injuries caused by laser lead extraction has been well documented [12, 2, 13, 14]. Between 1995 and 2008, the FDA Manufacturers and Use Defined Experience database found that approximately 70% of deaths during TLE were due to a vascular tear or laceration [14]. This requires urgent repair with hemostasis and may require cardiopulmonary bypass. Furthermore, rapid surgical intervention is key as irreversible neurological damage occurs within 5 to 10 min of cardiac tamponade [10]. In addition to the serious injuries being seen elsewhere in the literature, our conclusion that a cardiothoracic surgeon is still a vitally important member of the laser lead extraction team is also supported by the research of other groups [14–17]. It has been noted that the involvement of a surgeon may increase costs, but that this is outweighed by the benefit to the patient [17]. Given the potential morbidity and mortality the study supports continued CTS presence as backup for these cases.

This study is not without its limitations. This is a single center study which limits its generalizability, but it took place at a large academic medical center that serves a diverse community. Additionally, it is a retrospective study that involved chart reviews and is limited to the information available in the written record. Even given these inherent limitations to the study design, this study represents a large modern cohort and the low rates, but high severity of some of the complications seen in this study validate the continued need for cardiothoracic surgeons to be involved in the removal of cardiac leads.

Although the incidence of laser lead extraction complications that require surgical intervention seen in this study was relatively low, those complications were some of the most serious. Both incidences of injury requiring surgical intervention received it rapidly and were able to survive the index operation. Given these results, even with the advancement in technology making lead removal safer, having cardiothoracic surgery backup for laser lead extractions provides a necessary and lifesaving service.

## Data Availability

Upon requests.
